# Hospital Admission Factors Independently Affecting the Risk of Mortality of COVID-19 Patients

**DOI:** 10.3390/jcm12196264

**Published:** 2023-09-28

**Authors:** Marcin Paciorek, Carlo Bieńkowski, Justyna Dominika Kowalska, Agata Skrzat-Klapaczyńska, Agnieszka Bednarska, Dominika Krogulec, Grażyna Cholewińska, Jacek Kowalski, Regina Podlasin, Katarzyna Ropelewska-Łącka, Piotr Wasilewski, Piotr W. Boros, Magdalena M. Martusiewicz-Boros, Piotr Pulik, Andrzej Pihowicz, Andrzej Horban

**Affiliations:** 1 Department of Adults’ Infectious Diseases, Medical University of Warsaw, 02-001 Warsaw, Poland; mpaciorek@zakazny.pl (M.P.); jdkowalska@gmail.com (J.D.K.); agata.skrzatasw@gmail.com (A.S.-K.); abednarska@zakazny.pl (A.B.); dominika_krogulec@wp.pl (D.K.); ahorban@zakazny.pl (A.H.); 2 Hospital for Infectious Diseases in Warsaw, 01-201 Warsaw, Poland; cholegra@zakazny.pl (G.C.); jacekkowalski1982@gmail.com (J.K.); podlasin@zakazny.pl (R.P.); k.ropelewska@gmail.com (K.R.-Ł.); p.wasilewski@onet.eu (P.W.); ppulik@zakazny.pl (P.P.); apihowicz@zakazny.pl (A.P.); 3 Department of Infectious Diseases, Collegium Medicum, Cardinal Stefan Wyszynski University in Warsaw, 01-815 Warsaw, Poland; 4 Lung Pathophysiology Department, National TB & Lung Diseases Research Institute, 01-138 Warsaw, Poland; piotr.boros@gmail.com; 5 3rd Lung Diseases & Oncology Department, National TB & Lung Diseases Research Institute, 01-138 Warsaw, Poland; m.martusewicz@gmail.com

**Keywords:** COVID-19, SARS-CoV-2, mortality, risk factors

## Abstract

Introduction: COVID-19 is a disease characterized by high in-hospital mortality, which seems to be dependent on many predisposing factors. Objectives: The aim of this study was to analyze the clinical symptoms, abnormalities in the results of laboratory tests, and coexisting chronic diseases that independently affected the risk of in-hospital mortality in patients with COVID-19. Patients and Methods: We analyzed the records of patients with COVID-19 who were hospitalized from 6 March 2020 to 30 November 2021. Results: Out of the entire group of 2138 patients who were analyzed, 12.82% died during hospitalization. In-hospital mortality was independently associated with older age (OR 1.53, 95% CI 1.20–1.97); lower arterial blood oxygen saturation (OR 0.95, 95% CI 0.92–0.99); the presence of a neoplasm (OR 4.45, 95% CI 2.01–9.62), a stomach ulcer (OR 3.35, 95% CI 0.94–11.31), and dementia (OR 3.40, 95% CI 1.36–8.26); a higher score on the SOFA scale (OR 1.73, 95% CI 1.52–1.99); higher lactate dehydrogenase (LDH) (OR 1.08, 95% CI 1.05–1.12); higher N-terminal pro-brain natriuretic peptide (NT pro BNP) (OR 1.06, 95% CI 1.01–1.11); and lower total bilirubin in blood concentration (OR 0.94, 95% CI 0.90–0.99). Conclusions: We found that low oxygen saturation, old age, and the coexistence of cancer, gastric ulcers, and dementia syndrome were variables that independently increased mortality during hospitalization due to COVID-19. Moreover, we found that decreased platelet count and bilirubin concentration and increased levels of LDH and NT-proBNP were laboratory test results that independently indicated a higher risk of mortality. We also confirmed the usefulness of the SOFA scale in predicting treatment results. The ability to identify mortality risk factors on admission to hospital will facilitate both adjusting the intensity of treatment and the monitoring of patients infected with SARS-CoV-2.

## 1. Introduction

The COVID-19 disease caused by SARS-CoV-2 led to a pandemic in which millions of patients died. Due to the fact that a very large group of patients required hospitalization, the pandemic was also a great challenge for health care systems. In 2021, the number of deaths in Poland exceeded the average annual value measured over the last 50 years by nearly 154,000 [1].

The spectrum of severity of SARS-CoV-2 infection symptoms is highly diverse and includes asymptomatic or mild symptoms of upper respiratory system diseases, but also severe pneumonia with coexisting respiratory failure [2,3].

COVID-19 is a disease characterized by high in-hospital mortality [4] and the heterogeneity of the clinical course seems to be dependent on many predisposing factors [5].

The studies conducted show the relationship between the risk of death during hospitalization and the coexistence of certain chronic diseases, such as diabetes, obesity, hypertension, COPD, cancer, dementia, and congestive heart failure [6,7,8].

The results of numerous standard laboratory tests performed during hospitalization [9] may also be an important predictor of the course later taken by the disease. In addition, geographic differences in disease severity and mortality have been demonstrated in patients with COVID-19 [10], which clearly shows the need to identify independent risk factors worsening the prognosis in analyses carried out on a large number of patients in various geographical regions.

We conducted a single-center study on a large group of patients hospitalized due to COVID-19. The aim of this study was to analyze the clinical symptoms occurring on admission to the hospital, as well as the abnormalities in the results of initial laboratory tests, and the coexisting chronic diseases that independently affected the risk of in-hospital mortality for patients with COVID-19. Early identification of a group of patients with an increased risk of death would make it possible to identify indications for closer clinical monitoring and could have an impact on the course of hospitalization.

## 2. Methods

### 2.1. Study Design and Participants

We evaluated the records of adult patients (≥18 years old) diagnosed with COVID-19 who were admitted to the Hospital for Infectious Diseases in Warsaw from 6 March 2020 to 30 November 2021. The reason for hospitalization was respiratory failure, as well as complications of COVID-19 such as dehydration, impaired consciousness, and bacterial pneumonia. The diagnosis of COVID-19 was confirmed by testing nasopharyngeal swab samples using real-time reverse transcriptase-polymerase chain reaction (RT-PCR) or SARS-CoV-2 antigen tests. The exclusion criteria were missing data on both oxygen therapy on admission and arterial blood oxygen saturation.

All the patients had a definite outcome of hospital treatment: death or discharge from the hospital. If the patient was transferred directly to another hospital, the result of treatment was considered the outcome of the therapy administered in the hospital to which the patient was transferred.

### 2.2. Data Collection

Clinical data were collected by means of a questionnaire specifically designed for the purpose of this study, which included information about comorbidities, the results of physical examination, the methods applied, and the effects of treatment. Some of the questions concerning comorbidities were based on the Charlson Comorbidity Index [11], which is a simple standardized tool used to determine the risk of death associated with comorbidities; additionally, data on the coexistence of asthma, COPD, hypertension, alcohol abuse, and nicotine addiction were included in the questionnaire. Patients with diabetes requiring pharmacological treatment were divided into those with and without vascular complications. Moderate (creatinine concentration above 270 µmol/L) or severe (on dialysis, uremia, post-renal transplant) were considered factors indicating a prognosis for risk. A patient was considered to have liver disease if cirrhosis or chronic hepatitis were diagnosed. Alcohol abuse was qualified according to the CDC definition [12]. A standardized set of laboratory tests were performed. In addition, one of the parameters assessed was the SOFA score: a scale used in the definition of sepsis and the measuring of organ failure in sepsis, containing data on respiratory failure, hypotension, disturbances of consciousness, thrombocytopenia, renal failure, and increased bilirubin concentration. The data collected during the direct medical examination were verified by doctors caring for patients with COVID-19. After encoding and removing sensitive personal data, a specially trained group of doctors entered the data into a web application containing an electronic form adapted to the collection of data necessary for the implementation of the project. Data were stored in the application database in an anonymized, coded, and standardized manner. Laboratory results were imported into the application directly from the hospital information system.

### 2.3. Statistical Analysis

Univariate and multivariate logistic regressions were used to identify variables associated with the odds of death due to COVID-19. In the univariate model, variables were considered significant if the *p*-value for the Wald test was strictly below 0.05. The multivariate logistic regression model included those variables which were significant in the univariate model, except for those with the proportion of missing data exceeding 20%. All computations were performed using R version 4.1.1. (R Core Team, 2021, Vienna, Austria).

## 3. Results

The final analysis included 2138 patients hospitalized due to COVID-19 (1235 men and 903 women, median age 64, interquartile range [IQR] 50–74). Out of the entire group that was analyzed, 274 (12.82%) patients died during hospitalization and 1864 (87.18%) were discharged. Non-survivors were older than the patients who survived (median: 7.5 decades of life [6.7 to 8.4] vs. 6.2 decades [4.8 to 7.2], *p* < 0.001), as shown in Table 1. Moreover, patients who died during hospitalization had lower arterial blood oxygen saturation measured on admission to the hospital (median 85.0% [IQR 76.0–90.0] vs. median 93.0% [IQR 89.0 to 97.0], *p* < 0.001).

Non-survivor patients were more likely to present with some comorbidities and were less likely to be medical workers (Table 1). Furthermore, they scored higher on the SOFA score (median 3 [IQR 2–7] vs. median 1 [IQR 0–2], *p* < 0.001) on the day of admission to the hospital.

Analysis of laboratory parameters (Table 1) revealed many statistically significant differences between survivor and non-survivor patients, which are demonstrated in Table 2.

In the multiple logistic regression analysis (Table 3), in-hospital mortality was independently associated with older age (OR 1.53, 95% CI 1.20–1.97, *p* = 0.001); lower arterial blood oxygen saturation measured on admission to hospital (OR 0.95, 95% CI 0.92–0.99, *p* = 0.013); the presence of comorbidities: a neoplasm (OR 4.45, 95% CI 2.01–9.62, *p* < 0.001), a stomach ulcer (OR 3.35, 95% CI 0.94–11.31, *p* = 0.043), and dementia (OR 3.40, 95% CI 1.36–8.26, *p* = 0.008); a higher score on the SOFA scale (OR 1.73, 95% CI 1.52–1.99, *p* < 0.001); a higher lactate dehydrogenase blood concentration (OR 1.08, 95% CI 1.05–1.12, *p* < 0.001); higher NT-proBNP (OR 1.06, 95% CI 1.01–1.11, *p* = 0.033); and lower total bilirubin in blood concentration (OR 0.94, 95% CI 0.90–0.99, *p* = 0.010). 

## 4. Discussion

We conducted a single-center study analyzing the factors affecting in-hospital mortality in patients with COVID-19. In the large group of 2138 patients who were investigated, the in-hospital mortality rate due to multiple causes was 12.82%. The clinical course in patients hospitalized due to SARS-CoV-2 is highly diverse. In a systematic review and meta-analysis of 42 studies and more than 400,000 patients, the mortality rate ranged from a minimum of 3.14% to a maximum of 61.51%, with an overall mortality prevalence of 17.62% [13]. Such varied treatment outcomes may be influenced by many death-related risk factors observed on admission to the hospital.

In our study, age was one of the variables that had an independent effect on mortality (OR 1.53). This result is consistent with previous analyses, in which age significantly influenced the prognosis [13,14,15]. A probable explanation of this phenomenon can be found upon examining the differences in the functioning of the immune system in elderly people resulting in an inappropriate T cell response to infection, leading to a prolonged inflammatory stage [16]. Furthermore, aging individuals are characterized by a higher initial proinflammatory status with overactive cytokine production on the one hand and an impaired ability to have an effective immune reaction entailing efficient viral clearance on the other [15,17,18,19]. Old age, apart from influencing the host-dependent immune system response and consequently the clinical course of COVID-19, can obviously modify the course and exacerbate chronic diseases, which may increase the risk of complications not directly related to the infection.

Low arterial blood saturation measured on admission to the hospital was an independent factor increasing mortality (OR 0.95) in our investigation. Respiratory failure requiring oxygen therapy occurs in approximately 19% of the patients with a SARS-CoV-2 infection [5]. The occurrence of decreased blood saturation causes the hypoxia of tissues and organs and is associated with a worse prognosis [20,21].

Therefore, maintaining an adequate oxygen concentration in arterial blood is one of the main goals of therapy in patients infected with SARS-CoV-2. Moreover, arterial blood saturation is the main criterion determining the need for oxygen therapy and hospitalization, and also determines the indications for the use of many therapeutic options [22].

Among the comorbidities included in our analysis, the presence of dementia (OR 3.40), gastric ulcers (OR 3.55), and neoplasms (OR 4.45) was shown to have an independent effect on hospital mortality. The influence of comorbidities on the risk of death due to COVID-19 has been demonstrated in many studies. In a meta-analysis examining patients hospitalized due to COVID-19, the increase in mortality was shown to be related with the coexistence of COPD, diabetes, hypertension, cardiovascular diseases, and cancer [13].

In a study conducted in the UK, the effect of comorbidities on mortality was investigated in a large group of 20,000 hospitalized patients. Chronic cardiac disease, chronic non-asthmatic pulmonary disease, chronic kidney disease, obesity, chronic neurological disorder (such as stroke), dementia, malignancy, and liver disease independently increased the risk of hospital mortality [23].

As they are dependent on outside help, patients with lowered cognitive functions may not report early symptoms characteristic of COVID-19 [24,25], which may lead to complications such as dehydration.

In the investigation of a large community cohort of elderly patients of more than 500,000 volunteers aged >65 years old [26], coexisting dementia as compared to other comorbidities was associated with the largest increase in the risk of hospitalization due to COVID-19. Moreover, pre-existing dementia was also the strongest independent risk factor for hospital mortality.

In keeping with other studies [27,28], we also found the SOFA score to be an independent predictor of an unfavorable outcome in patients hospitalized due to COVID-19. The SOFA scale assesses the severity of organ failure. Its accuracy in assessing the risk of death during hospitalization has proved to be helpful in patients with sepsis [29,30].

Since severe COVID-19 meets the definition criteria and can therefore be regarded as viral sepsis, the usefulness of the scale and its impact on in-hospital mortality should not be surprising.

In our analysis, we showed the independent effect of several laboratory parameters on mortality during hospitalization. They were decreased platelet count and bilirubin concentration, as well as increased levels of lactate dehydrogenase (LDH) and N-terminal pro-brain natriuretic peptide (NT pro BNP). In the retrospective cohort study of Zeng et al. [31], laboratory parameters were measured on days 1–9, 10–15, and >15 from the onset of symptoms and LDH was determined to be independently associated with a fatal outcome: the odds ratios were greater than 5 in both the early and medium phases of COVID-19, together with age, elevated PCT, decreased lymphocyte count, and older age. In the systematic review performed by Izcovich and co-authors, in which 207 studies were included in the analysis, decreased platelet count led to a 14.3% increase in mortality with high-certainty evidence, high LDH levels caused a 10.4% increase in mortality with moderate-certainty evidence, and elevated brain natriuretic peptide caused a 12% increase in mortality with moderate-certainty evidence [27]. The prognostic value of NT-proBNP was demonstrated in the analysis of Gao and co-authors, with the best cut-off value predicting in-hospital death being 88.64 pg/mL, the sensitivity being 100%, and the specificity being 66.67% [32]. The analysis by Selcuk et al. also showed an independent effect of elevated NT-proBNP concentration on the increase in intra-hospital mortality in patients without heart failure who were hospitalized due to COVID-19 pneumonia [33]. Elevated NT-proBNP might be the result of direct cardiac injury, as demonstrated in the investigation by Shi and co-authors, which showed that patients hospitalized due to COVID-19 with an increased concentration of high-sensitivity troponin I and an increased level of plasma N-terminal pro-B-type natriuretic peptide had a higher risk of an unfavorable outcome [34]. In our investigation, the total bilirubin measured on admission was higher in patients with a fatal outcome as compared to patients who were discharged from the hospital (13.7 vs. 11.9 µmol/L, *p* = 0.007). Interestingly, in the multivariate logistic regression analysis, a higher concentration of bilirubin was associated with a favorable outcome (OR 0.94, CI 0.90–0.99, *p* = 0.010). In the analysis of Izcovich [27], which included 204 studies of high concentration of bilirubin in the blood, it was found to be one of the laboratory factors predisposing for poor prognosis, with a 12.6% increase in mortality and a low certainty of evidence.

In the large retrospective cohort study of Ding and co-authors [35], total bilirubin concentration on admission was higher in patients who passed away during hospitalization; however, after adjustments for other predisposing factors, only direct but not total bilirubin concentration was demonstrated to be an independent factor associated with an increase in hospital mortality.

Although this was not the subject of this analysis, when discussing the risk factors for the severe course of COVID-19, it is impossible not to mention the role of host genetic diversity that may modify its immune response [36], or host genetic differences regarding the angiotensin-converting enzyme-2 (ACE-2) to which the spike protein of SARS-CoV-2 binds to enter the host cells [37].

There were a few limitations of our study. Firstly, despite the implementation of standardized protocols for the assessment of the patient’s clinical condition and the application of a set of laboratory tests established for the purpose of study, it was a retrospective, single-center investigation. The retrospective nature of the analysis and the conditions related to the epidemic period resulted in the loss of a certain amount of data.

Treatment recommendations regarding indications for remdesivir or tocilizumab therapy, as well as the availability of these drugs, changed frequently. Our adoption of a 20% cut-off point in the amount of missing data for the variable to be included in the analysis implied the exclusion of the following: treatment with antibiotics and steroids, vaccinations against COVID-19, and the percentage of lung involvement by the inflammatory process shown in the performed chest CT scan. Finally, the time of our analysis covered the periods in which the prevalence of various forms of coronavirus mutation predominated, which could also have impacted the clinical picture.

## 5. Conclusions

In our analysis, we showed that low oxygen saturation on admission to the hospital, old age, and the coexistence of cancer, gastric ulcers, and dementia syndrome were variables independently increasing mortality during hospitalization due to COVID-19.

Moreover, we found that decreased platelet count and bilirubin concentration and increased levels of lactate dehydrogenase and N-terminal pro-brain natriuretic peptide measured on admission to the hospital were laboratory test results that independently indicated an increase in the risk of an unfavorable course of the disease. In addition, we also confirmed the usefulness of the SOFA scale in prognosing treatment results. The ability to identify mortality risk factors on admission to the hospital will facilitate the adjustment of treatment intensity and monitoring in patients infected with SARS-CoV-2. The intensified supervision of patients with clinical and laboratory risk factors could consist of more frequent or continuous (depending on the patient’s current condition) monitoring of saturation, respiration rate, periodic control of consciousness, and blood pressure, as well as performing control laboratory tests, in particular those enabling reassessment on the SOFA scale.

## Figures and Tables

**Table 1:** Univariate logistic regression analysis of factors associated with in-hospital mortality in patients with COVID-19. Demographics, clinical data, and comorbidities.

Variable	Total	Non-Survivors	Survivors	OR (95%CI)	*p*-Value
Gender, *n* (%)					
Female	903 (42.2)	115 (42.0)	788 (42.3)		
Male	1235 (57.8)	159 (58.0)	1076 (57.7)	1.01 (0.78–1.31)	*p* = 0.924
Age (decade) *	6.4 (5.0 to 7.4)	7.5 (6.7 to 8.4)	6.2 (4.8 to 7.2)	1.98 (1.78–2.20)	*p* < 0.001
Time pt 0 (days) *	25.4 (18.0 to 31.9)	28.2 (21.0 to 32.5)	24.9 (17.8 to 31.9)	1.02 (1.01–1.03)	*p* < 0.001
Time of onset (days) *	8.0 (6.0 to 11.0)	7.0 (5.0 to 10.0)	8.0 (6.0 to 11.0)	1.00 (0.99–1.01)	*p* = 0.467
SatO_2_ (%) *	93.0 (88.0 to 97.0)	85.0 (76.0 to 90.0)	93.0 (89.0 to 97.0)	0.89 (0.88–0.91)	*p* < 0.001
Dementia	111 (5.2)	41 (15.5)	70 (3.8)	4.70 (3.10–7.05)	*p* < 0.001
Stomach ulcer	60 (2.8)	13 (4.9)	47 (2.5)	1.96 (1.01–3.57)	*p* = 0.035
COPD	111 (5.2)	25 (9.5)	86 (4.6)	2.15 (1.32–3.37)	*p* = 0.001
Liver	50 (2.3)	6 (2.2)	44 (2.4)	0.95 (0.36–2.09)	*p* = 0.912
Diabetes ^#^	339 (15.9)	62 (23.0)	277 (14.9)	1.79 (1.30–2.43)	*p* < 0.001
Diabetes *	62 (2.9)	15 (5.6)	47 (2.5)	2.55 (1.35–4.54)	*p* = 0.002
Paresis	65 (3.1)	16 (6.1)	49 (2.6)	2.39 (1.30–4.17)	*p* = 0.003
Chronic renal failure	34 (1.6)	7 (2.6)	27 (1.5)	1.83 (0.73–4.02)	*p* = 0.159
Neoplasm	152 (7.2)	39 (14.7)	113 (6.1)	2.66 (1.78–3.89)	*p* < 0.001
Asthma	134 (6.4)	14 (5.3)	120 (6.5)	0.80 (0.43–1.36)	*p* = 0.434
Immunosuppression	61 (2.9)	16 (6.0)	45 (2.5)	2.55 (1.38–4.48)	*p* = 0.002
Interstitial lung disease	20 (0.9)	3 (1.1)	17 (0.9)	1.24 (0.29–3.72)	*p* = 0.733
Atrial fibrillation	191 (9.1)	59 (22.2)	132 (7.2)	3.69 (2.62–5.17)	*p* < 0.001
Hypertension	1042 (49.1)	176 (65.9)	866 (46.7)	2.21 (1.69–2.90)	*p* < 0.001
Alcohol abuse	72 (3.9)	11 (5.0)	61 (3.7)	1.35 (0.66–2.50)	*p* = 0.373
Nicotinism	443 (22.8)	66 (29.5)	377 (21.9)	1.49 (1.09–2.02)	*p* = 0.012
Medical worker	132 (6.5)	9 (3.6)	123 (6.9)	0.51 (0.24–0.96)	*p* = 0.055
BMI > 30 (kg/m^2^)	671 (31.4)	71 (25.9)	600 (32.2)	0.38 (0.14–1.19)	*p* = 0.066
BMI 18–30 (kg/m^2^)	1016 (47.5)	117 (42.7)	899 (48.2)	0.42 (0.16–1.29)	*p* = 0.093
BMI < 18.5 (kg/m^2^)	21 (1.0)	5 (1.8)	16 (0.9)	1.24 (0.33–8.08)	0.782
SOFA Score *	1.0 (0.0 to 2.0)	3.0 (2.0 to 7.0)	1.0 (0.0 to 2.0)	1.76 (1.65–1.89)	*p* < 0.001

Results are presented as odds ratio (OR) and 95% confidence interval (CI). * Data are presented as median (interquartile range) or n/N (%). Time pt 0: the time between the moment of admission of the analyzed patient to the hospital and the moment of admission of the first patient with COVID-19; time of onset: time from first symptoms to admission to our hospital; SatO_2_: arterial blood oxygen saturation measured on admission to the hospital; COPD: chronic pulmonary obstructive disease; liver: liver disease; diabetes ^#^: diabetes requiring pharmacological treatment without vascular complications; diabetes *: diabetes requiring pharmacological treatment with vascular complications; BMI: body mass index; SOFA: sequential organ failure assessment score.

**Table 2:** Univariate logistic regression analysis of factors associated with in-hospital mortality in patients with COVID-19. Laboratory blood analysis results.

Variable	Total	Non-Survivors	Survivors	OR (95%CI)	*p*-Value
CRP (mg/L) *	63.0 (34.0 to 160.0)	156.0 (63.8 to 215.0)	58.0 (30.0 to 149.0)	1.01 (1.00–1.01)	*p* < 0.001
PCT (ng/mL) *	0.1 (0.0 to 0.2)	0.2 (0.1 to 0.7)	0.1 (0.0 to 0.1)	1.06 (1.02–1.12)	*p* = 0.021
Il-6 *	43.2 (18.2 to 87.3)	99.5 (53.3 to 188.9)	37.6 (16.0 to 75.1)	1.00 (1.00–1.00)	*p* = 0.043
Neu (1000 cells/μL) *	4.8 (3.4 to 7.0)	6.6 (4.5 to 9.4)	4.7 (3.3 to 6.6)	1.17 (1.13–1.21)	*p* < 0.001
Lym (1000 cells/μL) *	0.9 (0.6 to 1.3)	0.6 (0.5 to 0.9)	0.9 (0.7 to 1.4)	0.47 (0.35–0.63)	*p* < 0.001
PLT (1000 cells/μL) *	214 (163 to 282)	199 (145 to 260)	216 (166 to 287)	1.00 (1.00–1.00)	*p* < 0.001
ALT (U/L) *	35.0 (23.0 to 56.0)	35.0 (23.0 to 54.0)	35.0 (23.0 to 57.0)	1.00 (1.00–1.00)	*p* = 0.344
eGFR ≥ 60 l/min/1.73 m^2^	1738 (82.3)	149 (55.6)	1589 (86.1)	0.20 (0.15–0.27)	*p* < 0.001
LDH (U/L) *	16.8 (12.2 to 22.5)	22.8 (16.8 to 32.3)	16.0 (11.8 to 21.4)	1.07 (1.05–1.08)	*p* < 0.001
Urea (mmol/L) *	5.7 (4.3 to 8.1)	9.4 (7.2 to 14.3)	5.5 (4.2 to 7.4)	1.18 (1.15–1.22)	*p* < 0.001
D-dimers mg/L *	2.1 (1.4 to 3.6)	3.6 (2.2 to 6.6)	2.0 (1.3 to 3.2)	1.04 (1.03–1.05)	*p* < 0.001
Bilirubin (μmol/L) *	12.1 (9.3 to 16.0)	13.7 (10.4 to 18.9)	11.9 (9.3 to 15.6)	1.01 (1.00–1.02)	*p* = 0.007
NT-pro-BNP (pg/mL) *	0.4 (0.1 to 1.5)	2.0 (0.9 to 6.0)	0.3 (0.1 to 1.2)	1.10 (1.07–1.12)	*p* < 0.001

Results are presented as odds ratio (OR) and 95% confidence interval (CI). * Data are presented as median (interquartile range) or n/N (%). CRP: C-reactive protein; PCT: procalcitonin; Il-6: interleukin 6; Neu: Neutrophils; Lym: Lymphocytes; PLT: platelets; ALT: alanine aminotransferase; eGFR: estimated glomerular filtration rate; LDH: lactate dehydrogenase; NT-pro-BNP: N-terminal fragment of B-type natriuretic propeptide.

**Table 3:** Multiple logistic regression analysis of factors independently associated with in-hospital mortality in patients with COVID-19.

Variable	*p*-Value	OR	95% CI
Age	0.001	1.53	(1.20–1.97)
Time pt 0	0.069	1.04	(1.00–1.10)
SatO_2_ (%)	0.013	0.95	(0.92–0.99)
Dementia	0.008	3.40	(1.36–8.26)
Stomach ulcer	0.043	3.55	(0.94–11.31)
COPD	0.432	1.50	(0.52–3.99)
Diabetes ^#^	0.837	1.07	1.07 (0.54–2.07)
Diabetes *	0.147	0.34	(0.07–1.35)
Paresis	0.546	0.67	(0.17–2.28)
Neoplasm	<0.001	4.45	(2.01–9.62)
Immunosuppression	0.280	2.56	0.37–12.16
Atrial fibrillation	0.542	1.29	(0.56–2.84)
Hypertension	0.689	0.89	0.50–1.58
Nicotinism	0.340	1.35	0.72–2.51
SOFA Score	<0.001	1.73	1.52–1.99
CRP	0.475	1.00	1.00–1.00
PCT	0.500	1.09	0.83–1.37
Il-6	0.119	1.00	1.00–1.00
Neutrophils	0.956	1.00	0.91–1.10
Lymphocytes	0.405	0.91	0.69–1.04
PLT	0.025	1.00	0.99–1.00
eGFR ≥ 60	0.590	1.23	0.58–2.71
LDH	<0.001	1.08	1.05–1.12
Urea	0.112	1.07	0.99–1.16
D-dimers	0.116	1.01	1.00–1.03
Bilirubin	0.010	0.94	0.90–0.99
NT pro BNP	0.033	1.06	1.01–1.11

Results are presented as odds ratio (OR) and confidence interval (CI). Time pt 0: the time between the moment of admission of the analyzed patient to the hospital and the moment of admission of the first patient with COVID-19; SatO_2_: arterial blood oxygen saturation measured on admission to Hospital; COPD: chronic pulmonary obstructive disease; diabetes ^#^: diabetes requiring pharmacological treatment without vascular complications; diabetes *: diabetes requiring pharmacological treatment with vascular complications; SOFA: sequential organ failure assessment score; CRP: C-reactive protein; PCT: procalcitonin; Il-6: interleukin 6; PLT: platelets; eGFR: estimated glomerular filtration rate; LDH: lactate dehydrogenase; NT-pro-BNP: N-terminal fragment of B-type natriuretic propeptide.

## Data Availability

The data sets used and/or analyzed during the current study can be made available by the corresponding author on reasonable request.
